# Microbial Metabolites in Colorectal Cancer: Basic and Clinical Implications

**DOI:** 10.3390/metabo11030159

**Published:** 2021-03-10

**Authors:** Yao Peng, Yuqiang Nie, Jun Yu, Chi Chun Wong

**Affiliations:** 1 Department of Gastroenterology, Guangzhou First People’s Hospital, Guangzhou Medical University, Guangzhou 510180, China; pennysmail@126.com (Y.P.); eynieyuqiang@scut.edu.cn (Y.N.); 2 Department of Gastroenterology, The Second Affiliated Hospital, Medical School, South China University of Technology, Guangzhou 510180, China; 3 Institute of Digestive Disease and Department of Medicine and Therapeutics, State Key Laboratory of Digestive Disease, Li Ka Shing Institute of Health Sciences, The Chinese University of Hong Kong, Hong Kong, China

**Keywords:** CRC, metabolomics, SCFAs (short chain fatty acids), bile acids, polyamines, clinical application

## Abstract

Colorectal cancer (CRC) is one of the leading cancers that cause cancer-related deaths worldwide. The gut microbiota has been proved to show relevance with colorectal tumorigenesis through microbial metabolites. By decomposing various dietary residues in the intestinal tract, gut microbiota harvest energy and produce a variety of metabolites to affect the host physiology. However, some of these metabolites are oncogenic factors for CRC. With the advent of metabolomics technology, studies profiling microbiota-derived metabolites have greatly accelerated the progress in our understanding of the host-microbiota metabolism interactions in CRC. In this review, we briefly summarize the present metabolomics techniques in microbial metabolites researches and the mechanisms of microbial metabolites in CRC pathogenesis, furthermore, we discuss the potential clinical applications of microbial metabolites in cancer diagnosis and treatment.

## 1. Introduction

Colorectal cancer (CRC) is one of the most common cancers and a leading cause of cancer-related deaths worldwide [1], which contributes the third most common diagnosis and second deadliest malignancy for both sexes. In developing countries, the incidence of CRC has been rapidly rising due to changes in diet and lifestyle [2]. For the past years, the incidence of new cases and mortality has been steadily declining, except for younger adults (younger than 50 years) [3]. CRC is mostly sporadic in nature, and colorectal tumorigenesis is a multistep process along an adenoma-carcinoma sequence over many years, which often involves a sequence of genetic and epigenetic alterations [4]. Nevertheless, environmental factors play important roles in modulating CRC development, as evidenced by epidemiologic studies showing that immigrants to western countries suffered an increase in CRC incidence as compared to their countries of origin [5,6]. Common environmental factors include dietary factors (red and processed meats, and alcohol), physical inactivity, environmental pollutants and gut microbiota dysbiosis [7,8]. These environmental factors could predispose individuals to greater risks of CRC, among which the role of microbiota dysbiosis has been increasingly appreciated [9].

Gut microbiota dysbiosis could be defined as abnormalities in the composition and function of the trillions of microbes (bacteria, fungi, viruses, and archaea) colonizing the intestinal tract, which represents the persistent departure from a healthy state to a disease-promoting state [10]. Generally speaking, this dysbiosis can be furthered distinguished into three separate categories, which often occur simultaneously: (a) depletion of commensal bacteria, (b) overgrowth of opportunistic pathogens potentially harmful microorganisms and (c) reduction in total microbiota diversity [11]. Mounting evidence suggests that gut microbiota dysbiosis was associated with colorectal tumorigenesis. Emerging studies revealed that specific pathogens and/or microbial communities play a key role in initiating or exacerbating tumorigenesis by inducing chronic inflammation, suppressing immunosurveillance and producing oncogenic metabolites [12]. For example, *Fusobacterium nucleatum* is highly enriched in colon tumor relative to adjacent normal tissue, suggesting that it may play a role in the development of CRC [13]. Functional and mechanistic studies supported that *F. nucleatum* promoted tumor development in the colon of *Apc*^min/+^ mice through the direct binding and activation of growth-promoting signaling cascades in cancer cells [14], as well as modification of the tumor microenvironment, such as the induction of a pro-inflammatory tumor milieu [15] and evasion of anti-cancer immune response [16]. Due to the presence of mucosal barrier along the intestinal tract, however, the direct contact between the gut microbiota with intestinal epithelial cells is limited. On the other hand, metabolites produced by gut microbiota are more readily translocated across mucosal barrier. Microbes may thus also promote cancer by producing metabolites that modulate cancer susceptibility or progression. It has become increasingly clear that the increase of gut microbiota-derived secondary bile acids, particularly deoxycholic acid (DCA), induces the development of CRC [17,18]. On the other side, the decrease of some beneficial microbial metabolites, such as butyrate, also plays a role in tumorigenesis [19].

With the advent of high-throughput metabolomic techniques, novel insights into host-microbiota metabolism interactions involved in colorectal tumorigenesis are being elucidated [20,21]. Together with metagenomic sequencing-based functional prediction, metabolomics-based profiling of microbial metabolites could reveal novel insights into the association with gut microbiota dysbiosis and the generation of detrimental metabolites that promote colorectal tumorigenesis. Conversely, the same approach could be utilized in the discovery of beneficial metabolites produced by intestinal commensals. In this review, we aim to provide an overview of metabolomic approaches to detect microbial metabolites, characterization of the metabolic pathways of microbial metabolites, and summarizing molecular mechanisms whereby microbial metabolites modulate CRC pathogenesis. In the end, we present studies that are relevant to the clinical application of microbial metabolites in CRC.

## 2. Measurement of Microbial Metabolites by Metabolomics

Metabolomics is defined as the study of small molecules involved in metabolic activity from biological specimens, including plasma, serum, urine, feces and tissue [22]. Our metabolome is the outcome of the extensive interactions between gene transcription, protein expression and environmental effects (e.g., gut microbiota). Hence, the detection of the metabolome provides a direct readout of host physiology [23]. As accumulating evidence indicates that gut microbiota modulates the development of CRC, at least in part, involves the generation of microbial metabolites, this rapidly developing technology has been applied to investigate host-microbiota interactions from the metabolic viewpoint. By combining with 16S rRNA amplicon sequencing or metagenomics, it offers a novel and powerful strategy to investigate and validate the collective metabolic activities of gut microbiota on dietary substrates and host intermediate products in CRC pre-clinical models. Furthermore, it also shows great potential in metabolite-based biomarkers screening for cancer diagnosis in clinical samples.

The two main methods applied in metabolomics are mass spectrometry (MS) and nuclear magnetic resonance (NMR) spectrometry. MS is becoming more widely used in host-microbiota research due to its high sensitivity, high-throughput, and applicability to a greater variety of metabolites. Due to the high complexity of biological specimens, MS analysis is usually coupled with gas or liquid chromatographic separation systems to improve the resolution of the specimens and the identification and quantification of subsequent metabolites [24]. Gas chromatography-mass spectrometry (GC-MS) is the most common method for short chain fatty acids (SCFAs) given their volatility in biological samples [25], but it is also used for many non-volatile metabolites, such as sugar and derivatives, amino acids and derivatives following chemical derivation steps [26]. Liquid chromatography-mass spectrometry (LC-MS) is even more widely used for the analysis of both non-polar metabolites (bile acids, lipids) [27] and polar metabolites (vitamins and their derivatives, amino acids, etc.) [28]. LC-MS employs softer ionization and lower temperature than GC-MS, making it more suitable for larger, non-volatile and less stable metabolites. Apart from MS approaches, a smaller number of studies utilized NMR for metabolomic profiling. However, NMR generally has lower sensitivity than MS-based methods, but it proceeds with relatively simple sample preparation. It also offers the advantage over MS-based methods as it provides valuable structural information, which is beneficial for identifying novel compounds [29].

Two complementary strategies are implemented for metabolomics analysis of microbial metabolites, involving global, untargeted profiling and targeted metabolites analyses. Untargeted metabolomics is the comprehensive analysis of all the detectable chemicals in a sample, where the tentative identification of thousands of compounds is based on database matching [30]. Both MS and NMR can be applied in untargeted metabolomics, however, the identification of the detected signals remains challenging in untargeted analysis. With the high diversity of microbial products, potentially many of the detected signals have not been previously characterized. Moreover, due to the wide concentration ranges of metabolites span over a dozen orders of magnitude, many metabolites such as SCFAs, amino acid, sugar and derivatives, cannot be precisely determined in one global untargeted metabolomics study. In that case, further targeted metabolomics approach complements the need to measure these relatively low abundance microbial metabolites such as SCFAs, bile acids and other small molecules, in addition to validation of the tentatively identified compounds by nontargeted metabolomics.

In recent years, advanced analytic platforms including desorption electrospray ionization mass spectrometry (DESI-MS) [31,32], matrix-assisted laser desorption ionization imaging mass spectrometry (MALDI-MS) [33] and nanoscale secondary ion mass spectrometry (NanoSI-MS) [34] have been developed to comprehensively enhance the resolution and metabolites coverage of conventional MS-based method. In addition, with the development of machine learning and artificial interagency, advances in computational methods have greatly assisted metabolomics data processing, metabolite identification, as well as in metabolic phenotyping and biomarker discovery [35,36].

In summary, both MS- and NMR-based approaches could be applied in metabolites analysis with their respective inherent advantages and disadvantages. The proper techniques should be selected according to metabolites of interest and specific sample type, and the instrument platform to be used in the study design step (Table 1). However, there is no bacteria-specific metabolomics method at present. The existing metabolomics methods detect the metabolites produced by both microbiota and host. By further intergrating with metagenomics, metatranscriptomics, and metaproteomics, it will facilitate to distinguish the microbial specific metabolites. Further experiments with germ-free animal models and specific genetic modified bacteria species help to explore the causality between microbial metabolites and diseases [37].

## 3. Function of Microbial Metabolites in CRC

**Figure 1:**
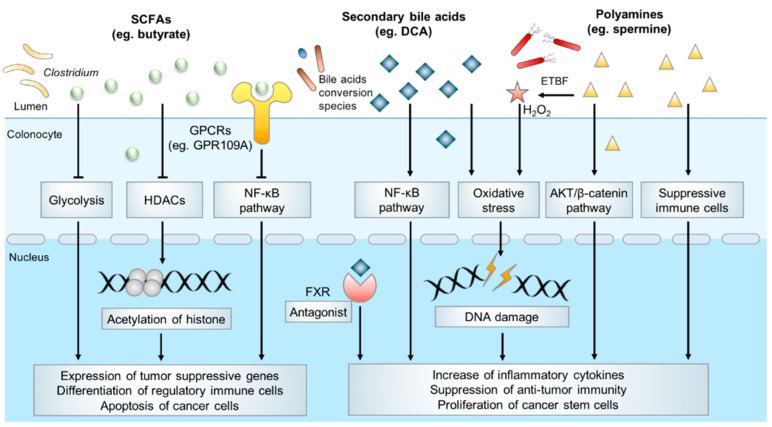
Typical microbial metabolites in CRC pathogenesis.

Microbiota-derived metabolites have a profound effect on host physiology, and it has been estimated that approximately 50% of all metabolites in the plasma have a bacterial origin [38]. With an estimated number of 10^14^ bacteria [39], the human colon harbors the densest and metabolically active microbial community in the body. Over the past decades, several catogries of gut microbial specific metabolites have been identified, including SCFAs, secondary bile acids, polyamines, indoles, methylamines, polyphenolics, vitamines and others [40]. Accumulating evidence indicates microbiota-derived metabolites exert an important influence on host physiology and diseases development. In the following parts, we will mainly summarize SCFAs, secondary bile acids, polyamine metabolism and their underlying molecular functions in colorectal carcinogenesis [41] (Figure 1).

### 3.1. Short-Chain Fatty Acids (SCFAs)

**Figure 2:**
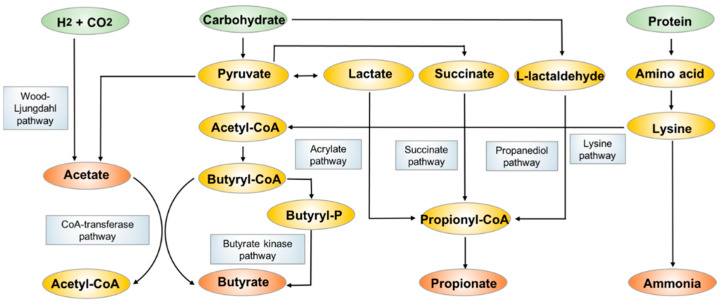
The biosynthesis and metabolism pathways of acetate, propionate and butyrate.

SCFAs are fatty acids with fewer than six carbon atoms, and they consist principally of acetate (C2), propionate (C3) and butyrate (C4). They are mostly produced from bacterial anaerobic fermentation of undigested dietary carbohydrates and endogenous epithelial-derived mucus [42] (Figure 2) in the colon. The most abundant acetate is produced by many gut commensal bacteria primarily through the fermentation of organic substrates, some acetogenic bacteria also generate acetate through the Wood-Ljundahl pathway [43,44]. Propionate is mainly produced by Bacteroidetes and some Firmicutes through the succinate pathway, while the other two pathways including the acrylate pathway and propanediol pathway also contribute to the formation of relatively smaller amounts of propionate [45]. Butyrate is formed by the most SCFA-productive microbial species in *Clostridium* and *Bifidobacterium*, dominantly using acetate as substate via butyryl-CoA:acetyl-CoA transferase activity [46,47]. The less commonly employed metabolic pathway is the butyrate kinase pathway [48]. Some bacterial species also undergo fermentation of protein and amino acids to generate butyrate through the lysine degradation pathway [49].

Acetate, propionate and butyrate typically have a combined concentration of 10–130 mM in the colon with a 3:1:1 ratio, which can be influenced by the gut microbiota composition, diet and other environmental factors. The concentration of SCFAs varies along the intestinal tract, with the highest levels in the cecum and proximal colon, and its levels decrease in the distal colon due to absorption by colonic epithelial cells. SCFAs are rapidly absorbed by colonocytes. In fact, butyrate is one of the chief energy sources for local colonic epithelial cells, while the majority of acetate and propionate enter the circulation to exert systemic effects, which influence various pathological conditions including obesity, fatty liver disease and metabolic syndrome [50,51]. Due to extensive absorption, only a small amount of unabsorbed SCFAs (about 5–10% of the total) are detected in fecal samples [52]. Hence, SCFAs could come directly into contact with the colon epithelium, and such interaction has received increasing attention due to the putative roles of SCFAs in colorectal tumorigenesis.

Butyrate is the most widely studied SCFA and it has been suggested that it plays a protective role in CRC [53]. Several clinical studies have reported the depletion of butyrate-producing bacteria species and diminished fecal butyrate levels are associated with colon tumorigenesis, suggesting that SCFAs may confer protective effects against carcinogenesis [54,55]. In homeostasis, butyrate helps to control the gut barrier function by providing colonocytes with energy, decreasing activity of type IV collagen to stimulate epithelial cell attachment and deterring the colonization of pathogens [46]. In CRC, butyrate inhibits tumorigenesis via directly inhibition of histone deacetylases (HDACs) activity to modulate translation of tumor suppressor genes. It also mediates its effect via alternative pathways such as metabolic rewiring of cancer cells, activation of G protein-coupled receptors (GPCRs) signaling pathways, which culminates in cancer cells apoptosis and anti-inflammatory responses [56].

Butyrate has a major impact on the epigenetic landscape in cancer cells, by virtue of its inhibitory effect on HDACs. In CRC, the dysregulation of HDACs creates a non-permissive chromatin conformation that prevents the transcription of tumor suppressive genes. HDACs inhibitors have shown great potential in anticancer therapy by reversing this process [57]. A growing body of work reported butyrate stimulates apoptosis of cancer cells through HDACs inhibition. In a gnotobiotic mice model treated with a butyrate-producing bacterium and high-fiber diet, the increased level of butyrate was associated with a lower tumor burden when compared with normal controls [58]. Furthermore, in CRC cell lines, butyrate was shown to induced expression of cell-cycle regulators such as p21 and p27 and pro-apoptotic genes such as FAS through inducing histone acetylation, thus to inhibit proliferation and promote apoptosis [59]. Butyrate also promotes an anti-inflammatory microenvironment, which is crucial for preventing tumorigenesis. By enhancing histone H3 acetylation and inhibiting the NF-κB signaling pathway, butyrate attenuated the production of inflammatory cytokines such as TNF-α, IL-6 and IL-12 to relieve colitis in mice model [60]. In addition, butyrate promotes regulatory T_reg_ cell differentiation [61] and inhibits macrophage pro-inflammatory function [62] through enhancing histone acetylation, thereby contributing to an anti-inflammatory microenvironment.

Apart from epigenetic modifications, butyrate also suppresses CRC development by modulating tumor metabolism. It has been well documented that butyrate serves as an energy source for normal colonic epithelial cells to sustain cell proliferation [63]. In contrast, butyrate induces metabolic rewiring in cancer cells to inhibit proliferation and induce apoptosis. For example, a study reported that butyrate could directly bind to PKM2, the M2 isoform of a pyruvate kinase, to inhibit aerobic glycolysis and reverse Warburg effects, thereby compromising the growth of cancer cells which are frequently dependent on glycolysis while normal cells are spared [64].

Butyrate is also an agonist of several GPCRs and regulates downstream signaling pathways. GPR109A is a typical receptor of butyrate. Activation of GPR109A induced down-regulation of Bcl-2, Bcl-xL, and cyclin D1, and up-regulation of death receptor pathway to promote apoptosis in colon cancer cells, together with the inhibition of NK-κB signaling [65]. In addition, GPR109A signaling induced by butyrate promotes the anti-inflammatory properties of macrophages and dendritic cells in colonic milieu to facilitate the differentiation of T_reg_ cells and IL-10-producing T cells [66]. Butyrate is also recognized by GPR43. By interacting with GPR43, microbial-derived butyrate suppresses the Wnt/β-catenin signaling to inhibit intestinal tumor development [67].

Although the majority of propionate is absorbed to enter the circulation and is metabolized in liver, it has also shown to have protective effects on colonic cells [68]. Propionate inhibits colonic inflammation [69,70]. Much less is known with regards to the molecular mechanism of propionate in colon cancer; however, it may share a similar mechanism with butyrate through inhibition of HDACs [71]. Acetate is also a potential chemopreventive agent against CRC by inhibiting cell proliferation and inducing apoptosis [72]. However, others reported that acetate promoted plasma membrane relocalization of MCT-1 and triggered increased glucose consumption and lactate production, thus increasing the glycolytic phenotype in cancer cells [73].

Based on current evidence, SCFAs are considered to exert preventive effects on CRC. More in-depth investigations will be required to understand its role in mediating colon epithelium and gut microbiota interactions in the context of tumorigenesis.

### 3.2. Bile Acids

**Figure 3:**
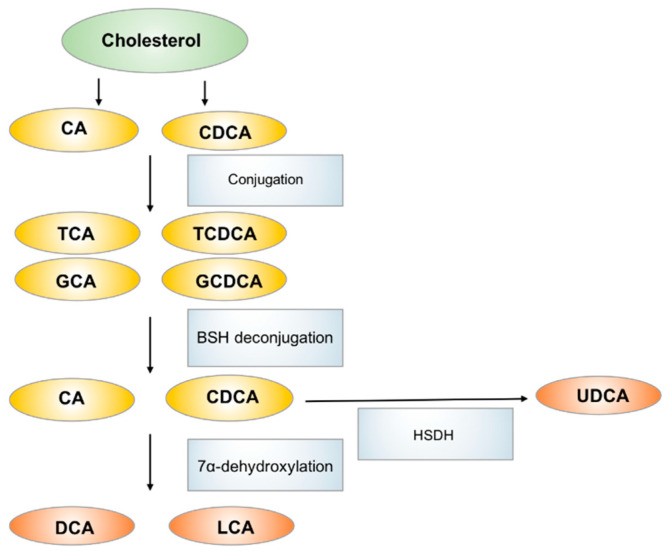
The biosynthesis and metabolism of bile acids in human. CA: cholic acid, CDCA: chenodeoxycholic acid, TCA: taurocholic acid, GCA: glycocholic acid, TCDCA: taurochenodeoxycholic acid, GCDCA: glycochenodeoxycholic acid, DCA: deoxycholic acid, LCA: lithicholic acid, UDCA: ursodeoxycholic acid.

Bile acids, which are primarily produced in the liver, are metabolized to secondary bile acids by the gut microbiota in the intestinal tract [74]. Primary bile acids, including cholic acid (CA) and chenodeoxycholic acid (CDCA), are produced from cholesterol in hepatocytes and are excreted through the bile duct after conjugation with glycine or taurine. The primary function of bile acid secretion is to emulsify lipids and to facilitate their absorption after meal ingestion. In the gastrointestinal tract, primary bile acids are mostly re-absorbed via enterohepatic circulation in the ileum. However, ~5% of bile acids are deconjugated and biotransformed to secondary bile acids via the action of gut microbiota in colon [75]. CA and CDCA are dehydroxylated to generate deoxycholic acid (DCA) and lithocholic acid (LCA) respectively. CDCA can also be metabolized by bacterial hydroxysteroid dehydrogenase (HSDH) to generate ursodeoxycholic acid (UDCA). The main bacterial genera involved in secondary bile acids biosynthesis are *Bacteroides*, *Clostridium*, *Lactobacillus*, *Bifidobacterium* and *Eubacterium*, they also serve key roles in regulating host fat metabolism [76]. The most abundant bile acids in humans include the primary bile acid CA and CDCA and the secondary bile acid DCA (Figure 3).

Metabolomics studies suggested a correlation between bile acid dysfunction and CRC in multiple cohorts. A multi-omics study combining metabolomics and microbiome analyses fecal/urinary metabolites and fecal microbiota revealed that the higher fecal concentrations of tumor-promoting DCA and increased levels of 7α-dehydroxylating bacteria were observed in CRC high risk population [77]. Another study also showed bile acids such as DCA were increased in multiple polypoid adenomas and intramucosal carcinomas patients, and were positively associated with the abundance of Bilophila wadsworthia, whose growth is stimulated by bile acids [78]. These studies indicate an extensive crosstalk between bile acids production and gut microbiota, which may form a positive feedback loop to drive CRC pathogenesis.

Secondary bile acids, especially DCA, are considered significant contributors to the development of CRC. DCA was first shown to be a carcinogen that promotes CRC development in mice in 1940 [79]. In a recent study, administration of DCA induced colonic tumor formation particularly in the context of obesity [80], as high-fat diet induced alterations in the gut microbiota contributed to increased intestinal DCA levels. Hydrophobic DCA exerts multiple pathogenic effects on the colon epithelium including the disruption of cell membrane, induction of oxidative damage to DNA, and activation of NF-κB to provoke inflammation. A study reports that DCA disrupted cell monolayer integrity and increased pro-inflammatory cytokine production in the intestine, leading to low grade inflammation and aggravation of intestinal tumorigenesis in *Apc*^Min/+^ mice [81]. In vitro experiments showed that DCA exposure induced single-strand breaks of DNA in CRC cells [82]. DCA-induced mitochondrial oxidative stress can activate NF-κB signaling in CRC cells, leading to decreased apoptosis and tumor progression [83]. Furthermore, bile acids could interact with bile acid receptors to promote CRC. DCA antagonizes intestinal farnesoid X receptor (FXR) function, which in turn, induces cell proliferation and Lgr^5+^ cancer stem cell expansion [84]. Collectively, DCA is a key secondary bile acid implicated in the promotion of colorectal tumorigenesis.

In contrast to DCA, UDCA and tauroursodeoxycholic acid (TUDCA) have been associated with suppression of colon tumor development. UDCA regulated intracellular reactive oxygen species (ROS) generation, suppressed cell cycle progression in colon cancer cells, and reduced the formation of colon cancer stem-like cells [85]. On the other hand, TUDCA suppressed NF-κB signaling in CRC cells and ameliorated colitis-associated tumorigenesis in AOM/DSS-treated mice [86]. The different biological and pathological effects of DCA and UDCA present are not been fully elucidated, one possible explanation for their different effects on cancer is the diverging effects on the same oncogenic signaling pathway [87]. These results suggest that the mechanisms by which secondary bile acids affect the progression of CRC are complex and more research is needed to fully understand their roles in colorectal tumorigenesis.

### 3.3. Polyamines

Polyamines are polycationic molecules that have more than two amino groups, and they are biosynthesized from the amino acids arginine and ornithine [88]. The intestinal tract contains high levels of polyamines, mainly including putrescine, spermidine and spermine, which are obtained from diet or biosynthesized by host and bacteria. Polyamine biosynthesis in the host involves arginase 1 (converts l-arginine to l-ornithine), the rate-limiting enzyme ornithine decarboxylase (ODC), which synthesizes putrescine from ornithine, and sequential enzymes catalyzing the interconversion of putrescine, spermidine and spermine. In contrast to host polyamine metabolism, bacteria use constitutive or inducible forms of amino acid decarboxylases to produce polyamines [89,90]. Polyamines are involved in a range biological processes. For the host, polyamines are essential to cell proliferation, immune cell differentiation and activation [91]. Several pathogens, such as *Escherichia coli*, *Helicobacter pylori*, and *Shigella flexneri* [92], also depend on polyamines for their virulence.

Polyamines are essential for cell growth, and the constrained intracellular polyamine level is linked to cell growth defects. As expected, tumor cells require more polyamines than normal cells to meet the demands for sustaining rapid growth, which is reflected in the increased levels of polyamines in urine or blood sample in cancer patients when compared to healthy individuals [93]. Dysregulation of polyamine metabolism by either the host and gut microbiota may thus be a contributing factor for CRC. A metabolomics screen comparing paired colon cancer and normal tissue samples from patients revealed that the host and microbiota participate in a positive feedback loop, whereby host CRC cells-generated polyamines promote bacterial biofilms growth, and in return, bacteria in biofilms generate polyamines to potentiate cancer development. Following treatment with antibiotics, resected CRC tissues harboring no biofilms or culturable bacteria had decreased levels of a polyamine metabolite, N^1^,N^12^-diacetylspermine, as compared to biofilm-positive tissues. Thus, host- and bacteria-derived polyamines may act synergistically to promote tumorigenesis [94].

Molecular mechanisms that are involved in polyamines toxicity are diverse. Pathogens induced polyamines catabolism, which generated a number of reactive toxic metabolites that could damage DNA, protein, and other cellular components. In the mice model of CRC, enterotoxigenic *Bacteroides fragilis* induced spermine oxidase (SMO) could catalyze spermine to spermidine and produced H_2_O_2_ as by-product, thus promoted intracellular oxidative stress, leading to DNA damage and accelerated carcinogenesis [95]. Polyamines also activate oncogenic signaling, as depletion of spermidine/spermine N^1^-acetyltransferase (SSAT) in CRC cells resulted in increased level of spermidine and spermine and the expression of pAKT and β-catenin and promoted cell proliferation and tumor metastasis [96]. Apart from tumor cell intrinsic effects, preclinical studies in mice indicate that polyamines suppress antitumor immune responses. Polyamine depletion through the inhibition of ODC activity could abrogate tumor growth in a T cell-dependent manner, which provided evidence that reducing intratumoral availability of polyamines could reverse immune suppression in the tumor microenvironment [97].

### 3.4. Other Microbial Metabolites in CRC

In recent years, the microbial derived indole and its derivatives have acquired a high notoriety due to their regulation of gastrointestinal barrier function and immune response. The microbial tryptophanase converts dietary tryptophan into indole, which is subsequently converted to various derivatives such as indole-sulfonic acid, indole-3-acetic acid, indole-3-propionic acid, etc. [98]. Many of indole derivatives are ligands of AhR, which plays a critical role in controlling the generation of immune cells at gut barrier site [98]. In a preclinical colitis mice model, oral administration of indole-3-pyruvic acid regulated T cell and dendritic cell function to make the anti-inflammatory milieu and ameliorated colitis [99]. These studies implied that indole derivatives may play a role in colon tumorigenesis. Another important class of microbial metabolites in the colon are methylamines. Previous epidemiological studies had provided evidence for a correlation between colorectal cancer and trimethylamine-N-oxide (TMAO), which is produced by the combined action of gut microbiota and hepatocytes from dietary choline and carnitine [100]. Multi-cohorts analysis also indicated microbial genes associated with trimethylamine synthesis enriched in CRC cohorts [101]. However, it remains elusive whether the increase in TMAO levels is a cause or a consequence of cancer. Recent studies indicated that TMAO could exacerbate chronic inflammation to promote carcinogenesis, additional studies are still needed to further validate the molecular mechanisms [102].

In summary, many microbial-derived metabolites profoundly affect colon tumorigenesis. Metabolites such as butyrate and indole-3-propionic acid may impose positive effects on cancer risks, while some other metabolites including DCA, spermine and TMAO increase cancer risk. Further exploration of the molecular mechanisms of microbial metabolites associated carcinogenesis are needed to validate the causality of these metabolites in CRC.

## 4. Implication for Clinical Applications of Microbial Metabolites in CRC

As shown by our summary above, huge strides have been made in our understanding of the role of gut microbiota-derived metabolites in health and disease, particularly for CRC. The discovery of microbial metabolites involved in colorectal tumorigenesis has key implication for the discovery of potential biomarkers for disease screening as well as novel therapeutic targets. Furthermore, the profile of gut microbiota and that of microbial metabolites is rapidly renewable varying with the diet, making it more amenable for therapeutic intervention in CRC progression. Elucidation of the role of microbial metabolites will provide a new paradigm for cancer diagnosis, prevention and treatment.

### 4.1. Use of Microbial Metabolites as Biomarkers

There are already various biomarkers available for non-invasive diagnosis of CRC. Fecal occult blood test (FOBT), serum tumor marker carcinoembryonic antigen (CEA) and methylated Septin9 are now commonly used biomarkers for CRC screening in the clinic [103,104]. Other molecular markers include microsatellite instability-high, BRAF and RAS mutations are predictive of patient prognosis and therapy response [105]. However, most of these biomarkers are significantly limited in clinical application due to their low sensitivity or specificity of detection. Hence, it is of great importance to develop accurate and non-invasive biomarkers for cancer screening and prognostication.

As mentioned above, some of the microbiota-derived metabolites are associated with CRC, and biomarker discovery from microbial metabolome is an area of active investigations. Several studies have found potential microbial metabolites as screening biomarkers in CRC cohorts. For instance, in a GC-MS based global stool metabolites profiling study, the higher level of acetate and the lower level of butyrate and UDCA were unveiled in CRC patients [106]. Another GC-MS metabolomic analysis using CRC tissue identified 19 differentiating metabolites, and pathway enrichment analysis revealed significant perturbation of short chain fatty acids metabolism, secondary bile acids metabolism and several carbohydrate metabolites pathways in CRC [107]. A paralleled investigation of tumor tissue and feces using NMR found the decreased level of butyrate in CRC patients, and the AUC for diagnosing CRC from normal samples in fecal and tissue sample were 0.692 and 0.717, respectively, and the level of fecal acetate demonstrated the highest diagnostic performance with an AUC of 0.843 [108]. A MS-based metabolomic analysis also discovered a panel of polyamine metabolites (N_1_-acetylspermidine, arginine, citrulline and ornithine) significantly upregulated in CRC cohort [109]. The integrated analysis of microbiome and metabolome revealed that the fecal abundance of microbial associated polyamines (putrescine and cadaverine) have high AUCs performance for CRC diagnosis [110]. These examples highlight the utility of metabolic markers for CRC screening.

Nevertheless, several metabolomics studies implied inconsistent findings. In a CRC cohort, the serum metabolomics analysis only detected serum glycochenodeoxycholate, a bile acid metabolite, that was positively associated with colorectal cancer among women, while no overall associations were observed between serum metabolites and CRC [18]. Meanwhile, in a fecal metabolomics study of SCFAs, the results demonstrated that SCFAs concentrations did not vary meaningfully between colonic adenoma, carcinoma and normal or after cancer treatment, indicating that fecal SCFAs are not predictive for colonic tumors [111]. These inconsistent results may due to the limited number of subjects in cohorts, different sample types and the use of different metabolomics analytic platforms and strategies. In summary, larger cohorts and standardized sample preparation and metabolomics analysis methods are needed to further evaluate the diagnostic value of microbial metabolites for CRC in clinical settings.

### 4.2. Modulation of Microbial Metabolites for CRC Prevention and Treatment

Cancer prevention represents an attractive strategy in reducing cancer burden. Epidemiological studies have indicated that dietary modulation is effective in reducing CRC risk [112]. The increased intake of red meat and processed meat is correlated with higher cancer risk, whereas dietary fiber may be protective. Diet profoundly affects the composition of microbial metabolites composition. In a cohort of advanced colorectal adenoma patients, a high-fiber diet subsequently increased microbial production of SCFAs, and was associated with a reduced cancer risk [54]. On the other hand, high-fat diet induced alterations in microbiota metabolite composition include increased secondary bile acids synthesis, decreased saccharolytic fermentation and butyrogenesis and colonic mucosa damage were also illustrated in another cohort with dietary exchanges experiment [113].

The direct supplementation of microbial metabolites has also shown promise in CRC prevention. Given the encouraging results in preclinical studies, butyrate supplementation could be a promising prevention strategy for CRC. In a short-term clinical trial (registered in the Australian New Zealand Clinical Trials Registry as ACTRN12609000306213), the supplementation of butyryated high-amylose maize starch in diet could significantly increase the SCFAs level and prevent the red meat-induced deleterious adduct formation in the rectum [114]. Furthermore, in preclinical patient derived CRC organoid models, butyrate was shown to improve the efficacy of radiotherapy, which suggests the potential clinical application of butyrate in combination with other cancer therapies [115]. There are also preclinical studies of butyrate to improve CRC surgery outcomes. The mechanical bowel preparation prior to CRC resection results in diminishment of SCFAs producing bacterial species and butyrate levels, which could result in impairment of the intestinal barrier, thus leading to bacterial translocation and possible infectious complications [116]. Evidence from rat models supported that oral or rectal administration of butyrate enhanced the bursting wall tension of anastomosis after left or right colectomy. Moreover, SCFAs could hinder the growth of pathogens related to anastomotic leakage [116].

As for polyamines, the prospective studies of dietary polyamines supplementation indicate that intake of polyamines above the median amount in the general population was associated with 39% higher risk of colorectal adenoma [117]. However, in another cohort of average-risk, postmenopausal women, dietary polyamines were not associated with increased risk of CRC or CRC-specific mortality [118]. More studies are needed to confirm the effect of dietary polyamines on CRC risk.

UDCA is a promising chemopreventive agent for CRC in several clinical trials [119,120]. It is reported that the participants taking UDCA were associated with a reduction in colonic neoplasia incidence. Interestingly, the beneficial effects of UDCA appear to be gender-specific, as a randomized clinical trial (registered with the FDA under Investigational New Drug No.50236) revealed that men treated with UDCA have reduced risk for developing advanced lesions, whilst women showed a significantly higher risk [121,122].

While there are only a few clinical trials of microbial metabolites to comprehensively evaluate the safety and efficacy of microbial metabolites at present, it provides a new paradigm in CRC prevention and treatment. Supplement of beneficial metabolites could be a feasible strategy to improve cancer therapy and surgery in the future.

## 5. Conclusions

With numerous metabolomic studies, changes in microbial metabolites and their functional role in CRC are beginning to be elucidated, and great progress have been made in our understanding of the molecular mechanisms of microbial metabolites in cancer. Microbial metabolites may impact the pathology of CRC by energizing host cells, modulating the host genome and regulating immune response. Despite being a relatively young field, emphasis now is gradually moving from descriptive studies of overall dynamic changes of microbial metabolites to the exploration of specific mechanisms involved in the pathogenesis of CRC. These studies also present unprecedented opportunities to develop novel clinical applications for CRC diagnosis and treatment. However, to make a more definitive statement regarding translational value of microbial metabolites, longitudinal prospective and large international cohorts are needed to validate these results. In conclusion, with these exciting developments, the study of microbiota-derived metabolites will provide more information on the gut microbiota-host metabolism interaction in the development of CRC. Furthermore, metabolomics studies enable the investigation of altered metabolites and impaired metabolic pathways during CRC treatment, thus defining the impact of peri-treatment management on the global metabolic status regarding host–microbiota interactions in CRC non-surgery and surgery therapy.

## Figures and Tables

**Table 1:** The common metabolomics methods in host-microbiome studies.

Strategies	Methods		Quantification	Metabolites Attributes	Advantages	Disadvantages	Prospects
Untargeted and targeted	NMR		Yes	Polar or non-polar	Simple sample prepration, structural information, identify novel compounds	Low sensitivity, poor selectivity, poor for quantification	Machine learning and artificial interagency to assist metabolomics data processing, metabolite identification, and biomarker discovery
	MS	GC-MS	Yes	Volatile metabolites or the volatile derivatives of metabolites	The most common method for SCFAs, good spectrum libraries	Relatively complex sample preparation, standards or/and database dependence	
		LC-MS		Polar or non-polar	Softer ionization and lower temperature than GC-MS to detect larger/non-volatile and less stable metabolites		
		DESI-MS		Broad metabolites particularly lipids	Rapid in situ assessment of metabolomic profiles		
		MALDI-MS		Complex sample and broad metabolites	Rapid and tolerant of impurities		
		NanoSI-MS		Stable-isotope labeled metabolites	Simultaneously identify and quantify metabolites in single cells		
